# Supplementary material for: Thin film lithium niobate on sapphire for integrated mid-infrared modulator
Source: Nat Commun. 2026 Feb 21;17:3050. doi: 10.1038/s41467-026-69880-5 (PMC13039532; doi:10.1038/s41467-026-69880-5)
Supplement: Supplementary file 1 — Supplementary Information [file 41467_2026_69880_MOESM1_ESM.pdf]

Thin film lithium niobate on sapphire for integrated mid-infrared modulator

Pierre Didier[1*], Prakhar Jain[1], Mathieu Bertrand[2], Jost Kellner[1], Oliver Pitz[1], Zhecheng Dai[3], Tristan Kuttner[1], Mattias Beck[2], Baile Chen[3], Jérome Faist[2], Rachel Grange[1]

[1]ETH Zurich, Department of Physics, Institute for Quantum Electronics, Optical Nanomaterial Group, Zurich, Switzerland.
[2]ETH Zurich, Department of Physics, Institute for Quantum Electronics, Quantum Optoelectronics Group, Zurich, Switzerland.
[3]School of Information Science and Technology, ShanghaiTech University, Shanghai 201210, China.

*Corresponding author(s). E-mail(s): pdidier@phys.ethz.ch;

Abstract

The mid-infrared spectrum, spanning from 3 to 14 µm, holds great promise for molecular spectroscopy and free space optical communication, benefiting from strong molecular absorption and reduced atmospheric attenuation. While progress in MIR photonics has accelerated due to improved sources and detectors, integrated low loss, high performance modulators remain limited. In order to address this gap, we demonstrate a broadband, high speed lithium niobate on sapphire Mach Zehnder electro optic modulator operating from 3.95 to 4.5 µm. The device shows a 3 dB bandwidth above 20 GHz, 17 dB extinction ratio, and $\boldsymbol{V_\pi L = 22}$ V·cm, with optical output power at the half milliwatt level. We demonstrate 10 Gbit s^{-1} data transmission and a 70 GHz frequency comb, uniquely combining integration, low propagation loss, extinction ratio and high-speed operation.

Supplementary Note 1: Device fabrication schematic

Supplementary Figure 1 shows a schematic representation of the fabrication process, as detailed in the Methods section, used to define photonic circuits in thin-film lithium niobate on sapphire.

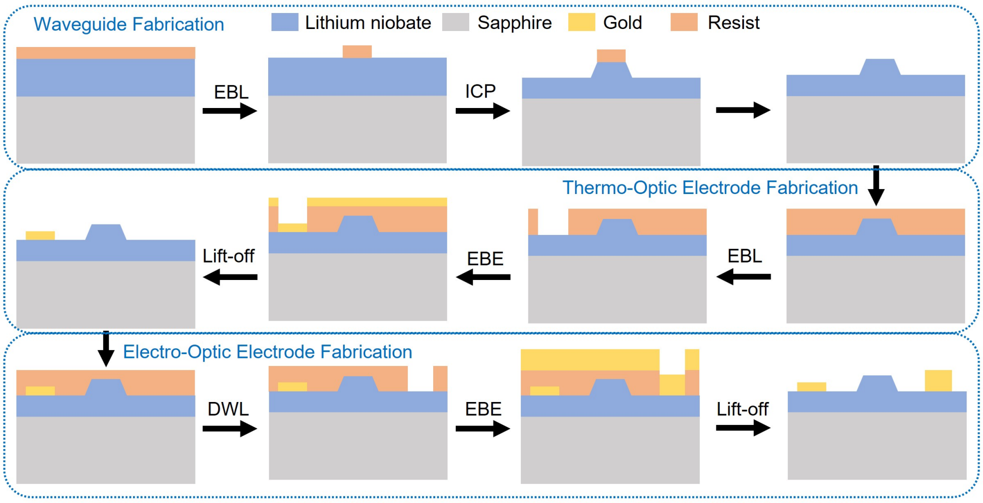

Supplementary Figure 1 Fabrication process of the TFLN on sapphire Fabrication process of the lithium niobate on sapphire. LNOS: Lithium niobate on sapphire. EBL: Electron beam lithography. ICP: Inductively Coupled Plasma. EBE: Electron beam evaporation. DWL: Direct-write laser lithography system.

Supplementary Note 2: Quantum cascade laser external cavity

A schematic representation of the setup is shown in Supplementary Figure 2a. The 4 µm quantum cascade laser was characterized in terms of output power, intensity, and voltage, exhibiting an output power of 20 mW at 0 °C with a corresponding bandwidth of approximately 150 nm, as shown in Supplementary Figures 2b and 2c, respectively.

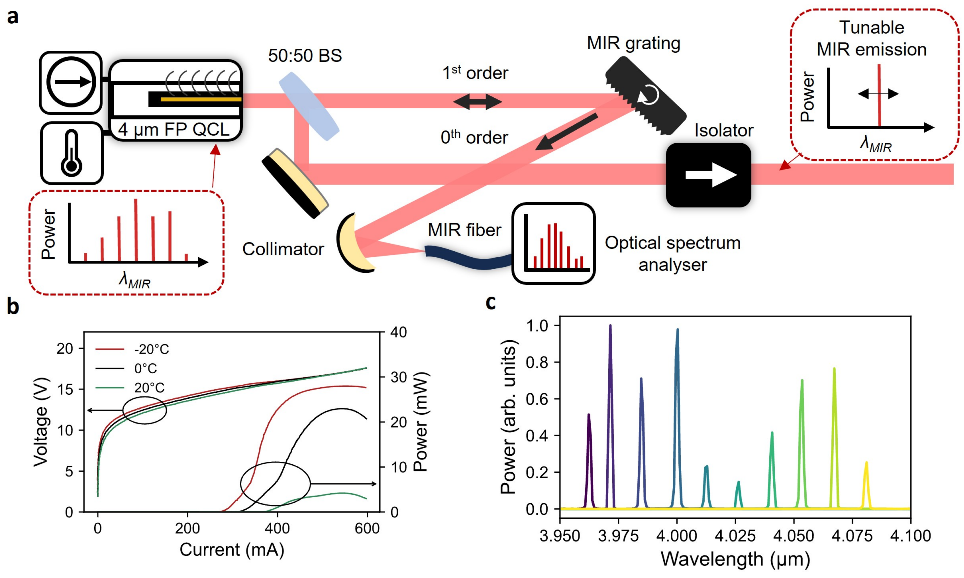

Supplementary Figure 2 Characteristics of the external-cavity quantum cascade laser at 4 µm a, Experimental setup of the external cavity configuration enabling the conversion from a multimode Fabry–Pérot emission at 4 µm to a tunable single-mode output. **b,** Power–voltage characteristics of the Fabry-Pérot quantum cascade laser used in the experiment. **c,** Emission spectra of the laser, demonstrating a tunability of approximately 150 nm.

Supplementary Note 3: Cutback measurement

The MMI performance was assessed using four-stage cascaded MMI structures. The result is highlighted in Supplementary Figure 3a. From the linear fitting of transmitted power, the excess MMI loss was found to be 0.52 dB at 4 µm and 0.19 dB at 4.3 µm, the latter being among the lowest reported values in this wavelength range. For the remaining characterization, light is coupled on and off the device using optimized linear grating couplers, ensuring consistent coupling conditions and improved measurement reproducibility. These couplers are first simulated using a commercial FDTD software, where parameter sweeps are performed to determine the optimal combination of grating period, duty cycle, and emission angle at the desired wavelength and polarization. To account for fabrication tolerances, an additional parameter sweep is conducted on fabricated test devices to identify the optimal design parameters. The scanning electron microscopy image in inset 1 of Supplementary Figure 3b illustrates the fabricated grating structure, showing well-defined teeth and uniform periodicity. The performance of the grating couplers was evaluated as a function of pitch distance for filling factors between 0.4 and 0.5, as shown in Supplementary Figure 3b. The minimum coupling losses were measured to be approximately 7.5 dB per grating at 4 µm and 8.84 dB at 4.5 µm. The grating performance suggests that this coupling

scheme could also be applied to the modulator, albeit at the cost of reduced wavelength tunability. Propagation losses were measured using waveguide lengths ranging from 0.3 cm to 3.5 cm as shown in Supplementary Figure 3c. The extracted losses are approximately 1.3 dB/cm at 4 µm, 4.4 dB/cm at 4.3 µm, and 5.3 dB/cm at 4.5 µm for a thin-film thickness of 1.5 µm and a single-mode waveguide. These values represent an upper bound on the intrinsic propagation loss since the bending effects were not included in the analysis. The similar losses at 4 µm and 4.3 µm confirm the suitability of the platform for modulator operation near 4.3 µm. An additional cutback measurement using on-fiber coupling allowed evaluation of the total in- and out-coupling losses, obtained from the extrapolated fit at zero length, which are 8.6 dB at 4 µm and 12.3 dB at 4.3 µm. Finally, power transmission through waveguides integrated with electro-optic electrodes was evaluated for electrode gaps ranging from 4.5 µm to 12.5 µm and for three different device lengths, as shown in Supplementary Figure 3d. These measurements enabled the estimation of plasmonic losses and provided justification for the selected electrode spacing used in the modulator design.

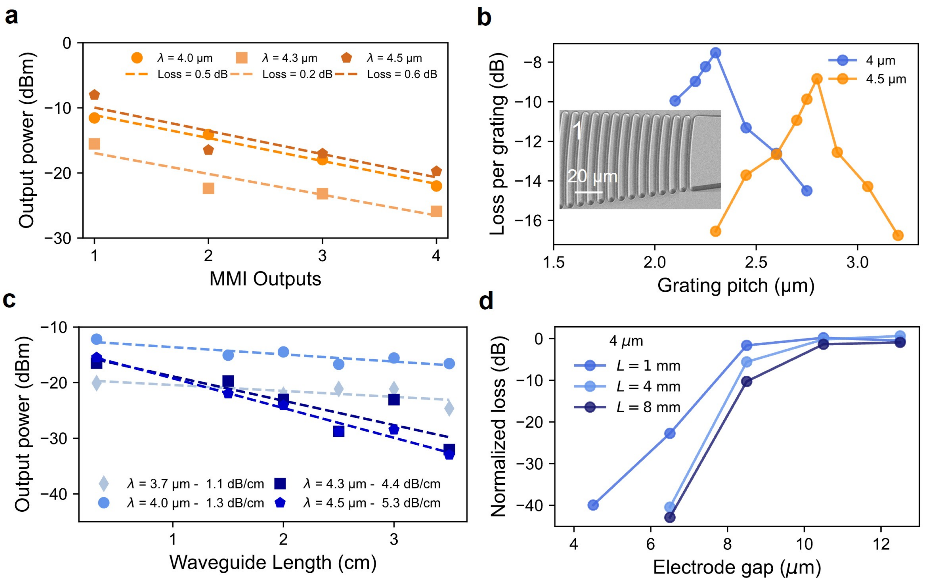

Supplementary Figure 3 Platform loss characterization. a, Evaluation of MMI loss using cascaded MMI structures. The slope of the linear fit provides the excess loss of the MMI at wavelengths of 3.7, 4.3, and 4.5 µm for waveguides with a top width of 2.5 µm and a thin-film thickness of 1.5 µm. **b,** Grating coupler loss as a function of pitch distance for filling factors between 0.4 and 0.5. The minimum loss at 4 µm is approximately 7.5 dB and 8.84 dB at 4.5 µm, respectively. The grating coupler was used for the cutback characterization and plasmonic loss evaluation to ensure consistent coupling conditions and improved measurement reliability. Inset 1 shows a scanning electron microscopy image of the grating coupler. **c,** Cutback measurements at wavelengths ranging from 3.7 to 4.5,µm using optimized grating coupler for in and out coupling, showing excellent linear fits and consistent power variation for propagation lengths between 0.3 cm and 3.5 cm. **d,** Power evaluation of waveguides with electro-optic electrodes for different electrode gaps from 4.5 µm to 12.5 µm and for three different lengths, allowing estimation of plasmonic losses and justification of the selected electrode spacing.

Supplementary Note 4: High speed characterization of InAs/InAsSb type-II superlattice photodetector

For optoelectronic characterization, the responsivity of the photodetector was measured using a Fourier transform infrared spectrometer (FTIR), calibrated with a blackbody source. As shown in Supplementary Figure 4a, the device exhibits a room-temperature responsivity of up to 0.8 A/W at 4 µm under bias voltages of 0 V, −0.5 V, and −1 V, with the responsivity approaching saturation beyond -0.5 V. For high-speed characterization, the bandwidth of the SMA-packaged photodetector was measured using a Lightwave Component Analyzer system, comprising a 67 GHz vector network analyzer (VNA) and a 1550 nm laser. Port 1 of the VNA was used to modulate the 1550 nm laser, while Port 2 was directly connected to the photodetector's SMA port to receive the electrical signal. The bias voltage was applied via a source meter through a

bias-tee. The parameter S_{21} of the device was extracted to obtain the bandwidth curve shown in Supplementary Figure 4b. The results reveal a bandwidth exceeding 15 GHz at bias voltages beyond -3 V, with a maximum bandwidth of approximately 20.3 GHz at -5 V, attributed to a slight inductive peaking effect from the wire bonding.

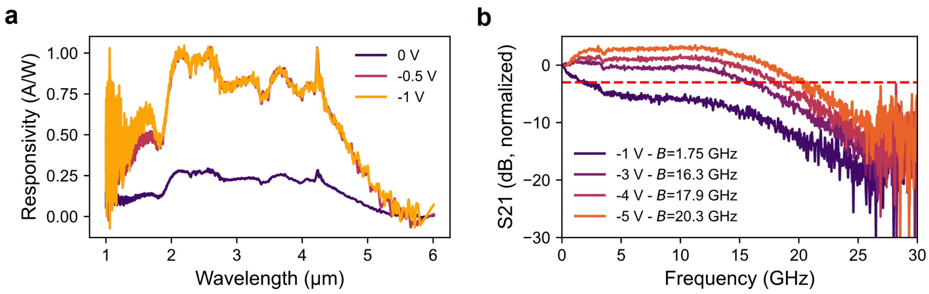

Supplementary Figure 4 High-speed electro-optic characterization of the MIR PD. a, Responsivity of the InAs/InAsSb type-II superlattice photodetector, reaching 0.8 A/W at 4 µm, characterized via FTIR measurements under a bias voltage of 0 V, -0.5 V and -1 V. **b,** Bandwidth of the whole high-speed packaged photodetector to an SMA port, exceeding a bandwidth of 20.3 GHz under bias voltage of -5 V, characterized via an LCA system.

Supplementary Note 5: High-speed design of the MZM modulator

The objective is to match both the impedance and the electrical refractive index to the optical group index. As shown in Supplementary Figure 5a, both parameters cannot be perfectly matched within the present geometry. Therefore, the preferred design prioritizes impedance matching to 50 Ω, since a slight offset in velocity matching only results in a limited bandwidth restriction. In this case, the velocity mismatch between the optical and electrical modes (2.33 versus 2.25 $\Delta n = 0.08$) and an electrode length of 1 cm would lead to a 3-dB bandwidth roll-off beyond approximately 188 GHz, which is not a significant limitation. To confirm the design based on the simulations, the measured S_{11} parameters for the modulators show return losses below −20 dB up to approximately 20 GHz, indicating excellent RF matching, as shown in Supplementary Figure 5b.

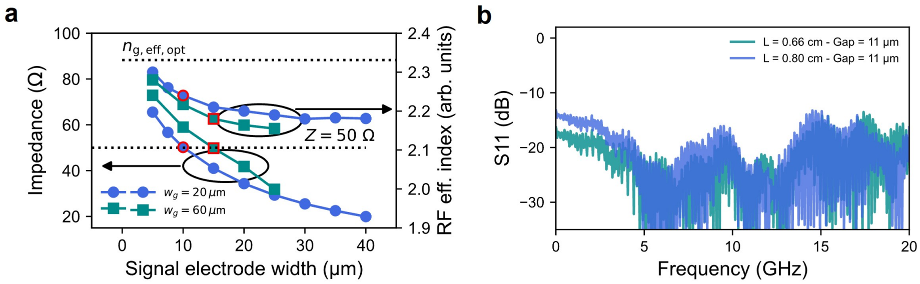

Supplementary Figure 5 RF design and impedance matching of the traveling-wave MZM a, Simulated impedance (left) and electrical refractive index (right) for the traveling-wave electrode geometry with two different electrode widths, 20 µm (blue) and 60 µm (dark cyan) for the 1.5 µm thin-film. The objective is to match the impedance to 50 Ω (lower black dashed line) and to align the electrical refractive index with the optical group index (upper black dashed line). Two designs were tested for comparison, in particular to assess whether a slightly wider signal electrode could reduce propagation loss. The point highlighted in red shows the design choice for the device. **b,** Measured S_{11} parameters showing the reflected electrical power as a function of frequency. The modulators exhibit return losses below –15 dB up to approximately 20 GHz, indicating excellent RF matching. The two designs, corresponding to $w_g = 20$ µm with $w_s = 20$ µm (blue) and $w_g = 60$ µm with $w_s = 15$ µm (dark cyan) show only minor differences in performance.
